# Molecular pathogenesis, diagnosis, and management challenges in complete androgen insensitivity syndrome

**DOI:** 10.3389/fendo.2025.1600343

**Published:** 2025-10-14

**Authors:** Chunqing Wang, Qinjie Tian

**Affiliations:** ^1^ Department of Ultrasound, Beijing Tiantan Hospital, Capital Medical University, Beijing, China; ^2^ Department of Obstetrics and Gynecology, Peking Union Medical College Hospital, Peking Union Medical College, Chinese Academy of Medical Sciences, Beijing, China

**Keywords:** complete androgen insensitivity syndrome, disorders of sex development, androgen receptor, gonadal development, sex determination, sex assignment, sexual differentiation

## Abstract

Complete androgen insensitivity syndrome (CAIS) is a rare X-linked recessive disorder of sex development (DSD) caused by androgen receptor (AR) gene mutation and present with female phenotypes with male chromosomal karyotype. Primitive bipotent gonads in CAIS differentiate into testes producing androgens and antimüllerian hormone (AMH). However, androgens cannot stimulate embryonic wolffian ducts into male internal reproductive organs owing to AR defect and hormone resistance, while AMH induces the regression of müllerian ducts with the absence of uterus, fallopian tubes, and upper third of the vagina. Thus, with male sex chromosome and testes, individuals with CAIS present with a typical female phenotype, primary amenorrhea (PA) and infertility, spontaneous thelarche during puberty, absent or sparse axillary/pubic hair, and increased risk of gonadal tumors in cryptorchidism. Though theoretically CAIS can be screened prenatally through a discrepancy between chromosomal karyotype and fetal external genitalia, suspected in bilateral inguinal “hernia” cases with female genital phenotype, and considered in cases with elevated testosterone (T) levels but no signs of virilization, the lack of typical symptoms brings great challenges to diagnosis and management. Endocrinological hormone assay is helpful for the identification of CAIS which reveals normal or elevated T levels, elevated luteinizing hormone for impairment of negative feedback of T, and normal follicle-stimulating hormone which is regulated by both sex hormones and inhibin. The diagnosis of CAIS after puberty is similar to the diagnostic workflow of PA with additional tests and should be differentiated with PA-related etiologies and other kinds of DSD, such as Swyer syndrome, Mayer–Rokitanskey–Küster–Haüser syndrome, Leydig cell hypoplasia, and several steroidogenic enzymatic deficiencies. Clinical manifestations, hormonal profiles, chromosomal karyotype, and pelvic imaging can provide comprehensive information for diagnosis. AR gene test or binding capacity can be performed for definitive diagnosis. The management of CAIS includes gonadectomy, hormone supplementation, and psychological support and education. Although with the development of molecular biology and awareness of the clinical entity more cases were reported, diagnostic and management challenges exist due to the disease-related and treatment-related stress including the rarity, untypical clinical manifestations, increased risk of gonadal malignancy, and its influence on physiology and psychology. This review provides a comprehensive overview of the molecular pathogenesis, pathophysiology, diagnostic evaluation, differential diagnosis, and management of CAIS.

## Introduction

Androgen insensitivity syndrome (AIS) (OMIM#300068) is a rare X-linked recessive disorder caused by mutations in androgen receptor (AR) gene located on chromosome Xq1.1-1.2, which results in impairment of pre- and postnatal masculinization and female phenotype albeit with a male chromosomal karyotype (1–3). Although considered rare, AIS is the most common disorder of sex development (DSD) in 46, XY individual, accounts for 0.8%–2.4% of phenotypic female cases with bilateral inguinal hernia, and is the third cause of primary amenorrhea (PA) (4–7). AIS refers to a wide spectrum of phenotypes associated with AR mutations from a completely female phenotype, ambiguous genitalia, to a typically male phenotype. Androgens are key elements in male sexual differentiation and cannot play their role in central and peripheral target organs and lead to complete female genitalia, various degrees of atypical genitalia, or nearly normal male phenotype as a result of AR mutation (8). Based on the severity of impairment of AR function, AIS can be divided into three subtypes: complete AIS (CAIS) with loss of function AR mutation, partial AIS (PAIS), and mild AIS (MAIS) with maintenance of AR function to distinct degrees. Although CAIS, PAIS, and MAIS all result from the identical *AR* gene mutation, their external genital phenotype, sex determination, pubertal development, presence or absence of cryptorchidism, and management are quite different (9). This review focuses on CAIS with complete androgen resistance, the most severe type of AIS based on residual AR activity.

Common complaints of CAIS can be inguinal mass, PA, and primary infertility which negatively impact patients’ physical and psychological condition and their quality of life (QOL). Imaging findings of CAIS present with the presence of undescended testis and neither wolffian duct derivative structures including epididymis, vas deferens, and seminal vesicles or müllerian structures such as uterus and upper third of the vagina (10–12). Although with the development of genetics and molecular biology and awareness of the clinical entity definitive molecular diagnosis can be realized in more than 90%–95% CAIS cases, diagnostic and management challenges exist due to the disease-related and treatment-related stress including the rarity, untypical clinical manifestations, increased risk of gonadal malignancy, and its influence on physiology and psychology (13–15). This article summarizes current fundamental and clinical aspects of CAIS to help understand the pathogenesis, accurately diagnose and differentiate similar disorders of DSD, and provide optimal, precise, and individualized management.

## Epidemiology

AIS, first described as syndrome of testicular feminization in a male pseudohermaphrodite by an Italian obstetrician in the1950s, is referred to the continuous clinical spectrum, and the underlying molecular etiology was not revealed until 20 years later with the discovery of *AR* (16, 17). CAIS accounts for approximately 1 in 20,400 to 1 in 99,100 in 46, XY males in western countries (18).

## Normal human gonadal development

CAIS is a type of congenital DSD, so understanding the normal embryonic development of genital organs and sexual differentiation is of significance for clinical reasoning in DSD including CAIS. Human gonadal differentiation and development is a complex process involving sexual chromosomes, multiple genes, and signaling pathways with comprehensive spatiotemporal map (19). Embryonic primordial gonads are bipotent before 6 weeks of gestation, with two independent reproductive systems of the wolffian ducts evolving into male genital organs and the müllerian ducts differentiating into female internal reproductive structures, which depend on the differentiation of primitive gonads—for instance, primordial cells migrate into the germinal ridge of thickened coelomic epithelium and induce the formation of testicular cord at 6 weeks of gestation, which initiates male gonadal development (20). Hence, sex determination is the initial step involved in the complex cascade of genetic and physiological events in the formation of typical male and female external and internal genitalia (21).

## Normal male sex determination and sexual differentiation

**Figure 1:**
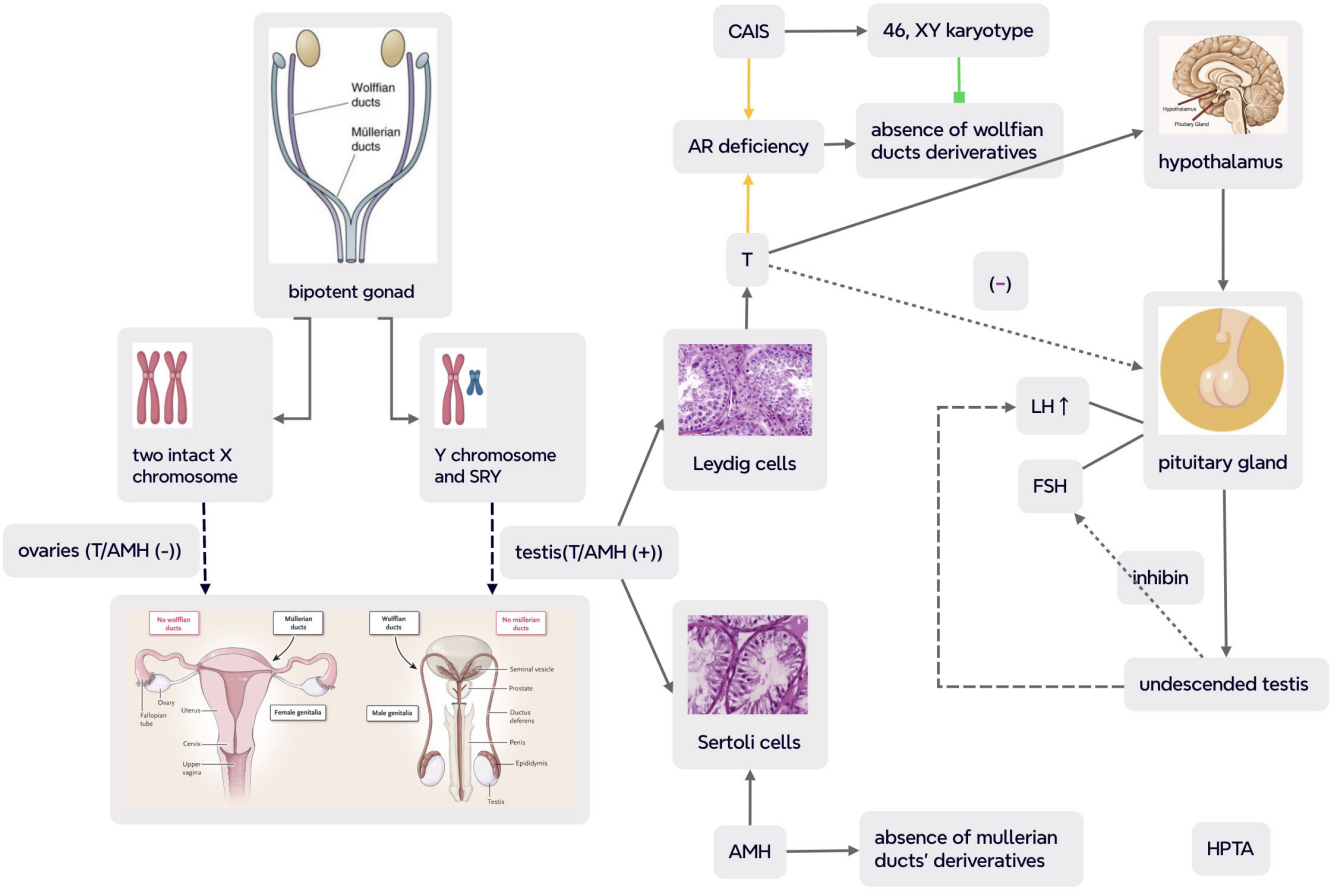
Normal human gonadal differentiation and developmental process and pathogenesis and pathophysiology in CAIS (complete androgen insensitivity syndrome) (part of the scheme is from (24)). In CAIS with complete defect in androgen receptor (AR) action, albeit with functional testis, the interaction between androgens and AR was disrupted and the transcriptional activity of the androgen–AR complex was reduced with undeveloped wolffian ducts and interference of masculinization. Meanwhile, müllerian ducts were also suppressed as a result of AMH secretion by testicular mesenchyma. So even with male chromosomal karyotype and gonads, cases with CAIS present a female phenotype, primary amenorrhea and infertility with absence of uterus, fallopian tubes, and upper third of the vagina. Negative feedback of T on hypothalamic–pituitary–testicular axis (HPTA) leads to an overproduction of pituitary LH and normal synthesis of FSH which is mainly regulated by inhibin. The dash line indicates impaired feedback of testicular T on pituitary gonadotropin in CAIS. CAIS, complete androgen insensitivity syndrome; SRY, sex-determining region Y; T, testosterone; AMH, antimüllerian hormone; LH, luteinizing hormone; FSH, follicle-stimulating hormone; HPTA, hypothalamus–pituitary–testicular axis.

The presence or absence of Y chromosome plays critical roles in sex differentiation, and embryos with a Y chromosome commonly become male individuals (22). The presence of Y chromosome, especially the expression of *SRY* gene (a major testicular determining factor) which maps to Yp, leads the undifferentiated primitive gonads into testes from the genital ridge (23). Two types of testicular parenchymal and mesenchymal tissues secret testosterone (T) (by Leydig cells) and antimüllerian hormone (AMH) (by Sertoli cells), respectively. T stimulates the development of wolffian ducts into the vas deferens, epididymis, and seminal vesicles of male phenotype, while AMH induces the regression of müllerian ducts (as illustrated in **Figure 1**) (24). 5-alpha dihydrotestosterone (DHT), a derivative of T, mediates genital tubercle to male external genitalia. It is worth noting that both kinds of androgens must bind to the single AR to exert their modulating role—that is, both the role of T on wolffian-duct-derived structures and the action of DHT on genital-tubercle-derived structures depend on a single intracellular functional AR (25, 26).

## Normal female sex determination and sexual differentiation

The absence of *SRY* gene and the presence of two intact X chromosomes mediate primitive gonads into ovaries. Without stimulation of T action, wolffian ducts will be degenerated, while müllerian ducts differentiate into uterus, cervix, fallopian tubes, and upper vagina due to lack of AMH (24). However, ovarian differentiation is not a passive process, and the development of normal female reproductive organs involves the expression of multiple genes and the regulation of signaling pathways (27).

## Sex determination and sexual differentiation in CAIS

An individual with CAIS has 46, XY karyotype, so the primitive genital ridge differentiates into testes producing T and AMH. The secretion of AMH by Sertoli cells results in the regression of müllerian ducts and the absence of uterus, cervix, and upper vagina. Meanwhile, albeit with T synthesis by Leyidg cells, wolffian ducts cannot develop and mature due to lack of functional AR and consequent androgenic actions. As a result of AR defect, DHT cannot stimulate male external genitalia either. Therefore, with 46, XY chromosome, testes, and male sex hormones, CAIS cases present with a female external genital phenotype with the absence of both wolffian and müllerian duct derivatives.

## Common human gender assignment

It is frequently not difficult to identify gender assignment in a 46, XX individual with a female phenotype or a 46, XY male phenotype. These are commonly consistent in each individual, such as 46, XX women with ovaries and female genitalia and 46, XY men with testis and male genitalia. Moreover, personal psychological gender is unchanged in normal male or female persons throughout life in the majority of individuals. As a matter of fact, human gender includes chromosomal, gonadal, anatomical, phenotypic, and psychological aspects, and the development of sexual identity beyond its biological determination is internal and complex (28). Inconsistency among chromosomal, gonadal, and phenotypic genders leads to DSD and may bring challenges to both the patients and their families. In patients with DSD, gender assignment can become a dilemma especially if inappropriate gender was determined in cases with ambiguous genitalia at birth which needed to be modified afterwards (24, 29, 30).

## Genetic etiology and molecular pathogenesis of CAIS

CAIS is caused by *AR*, the only X-chromosomal steroid receptor gene located on Xq1.1-1.2 gene mutation.

Understanding the structure and function of AR is important for an accurate diagnosis of CAIS, PAIS, and MAIS (31). As mentioned above, androgens, mainly T and DHT, play important roles in the expression of male phenotype, including male sexual differentiation, maintenance of secondary male characteristics, and initiation of spermatogenesis by binding to AR and forming a T/DHT-AR complex. The *AR* gene comprises eight exons and encodes a ligand-dependent protein of a member of transcription factor nuclear receptor superfamily consisting of 919 amino acids with a molecular weight of 110 kDa and three functional domains (N-terminal transactivation domain (NTD), DNA binding domain (DBD), and ligand binding domain (LBD)) and a helix region (32–35). Transcription activation of AR involves two activation functional regions (AF): AF1 in NTD encoded by exon 1 and AF2 in LBD by exon 3–8. The median DBD, encoded by exon 2 and 3, encompasses two zinc protein modules which mediate contact, interaction with DNA, and DNA dimerization process and are impacted by the interaction between cysteine and zinc ion (13).

Now it is confirmed that defects in AR interfere with normal male external and internal genital development in 46, XY individuals (33). The disruption of DNA or steroid binding domains was frequently associated with CAIS, but none in the helix region (36). AR gene mutations were also described in other diseases such as prostate cancer and Kennedy’s syndrome (spinal and bulbar muscle atrophy), and discoveries of such mutations also shed light on the importance of AR to reproductive biology, development, and tumorigenesis (36, 37). Furthermore, 47.6% AR mutations were located in LBD in a single-center study of 21 CAIS cases with cytogenetic diagnosis (38). Up to more than 900 AR mutations were found to be associated with disorders from approximately 150 reports in the last two decades such as *AR* single point mutation, leading to amino acid substitutions or premature stop codons, insertion, or deletion resulting in a frame shift mutation, complete or partial gene deletion, and intronic mutation causing mRNA splicing alteration (13, 39–43). These mutations result in conformational or major structural changes of the encoded polypeptide including truncated protein. Target organs showed no response to T with the absence of T action due to mutant *AR* and defective AR function, although normal or even elevated T levels were detected (35).

## Pathophysiology

Male sexual differentiation is mainly induced by T/DHT and hypothalamus–pituitary–gonadal axis (HPGA). AR is expressed in arcuate nucleus (ARC) kisspeptin neurons of the hypothalamus which play important roles in sex development. Normally, kisspeptin from ARC positively mediates hypothalamic gonadotropin-releasing hormone and pituitary luteinizing hormone (LH) pulsatile secretion, and androgen suppresses the production of kisspeptin (44–46). Complete androgen resistance in CAIS results in agenesis of wolffian ducts in the embryonic period and impairment of negative feedback of T on pituitary gonadotropin during adolescence and adulthood. Therefore, albeit with an elevated basal T level, LH level is yet upregulated, while follicle-stimulating hormone (FSH) concentration may be within the normal range which may be explained by the fact that LH is solely mediated by sex hormones and FSH is mainly regulated by gonadal inhibin (4, 24, 47) (as demonstrated in **Figure 1**). With lack of functional AR and T action, axillary and pubic hair is absent or sparse during puberty, but E derived from the aromatization of T can stimulate thelarche, while the absence of a uterus leads to absolute uterine PA and infertility. Dysregulation of HPGA and abnormality of the external genitalia may contribute to cryptorchidism at various descent stages from intraabdominal testis to testis in the inguinal canal or labioscrotum.

## Clinical manifestation

In infancy and childhood, the early clinical manifestation may be inguinal hernia or labial swelling (13). The primary clinical presentation of CAIS during puberty can be PA (48). During adulthood, infertility may be the complaint. Occasionally, the patient may present with pain in the inguinal region and be misdiagnosed with strangulated femoral hernia on imaging findings (49). CAIS patients may be taller than the common female population (>90% percentile) as a result of unaffected growth-controlling gene located on Y chromosome (GCY) (13, 24). Osteopenia and osteoporosis may occur in CAIS cases after the removal of testis (50).

Clinical signs: On physical examination, breast development at different stages can be observed. The external genitalia presents as female phenotype with absence or sparse pubic hair. Vaginal length may be normal or short with blind ending without cervix and uterus, and vaginal length can be used as an adjunctive screening tool for CAIS (7). Cryptorchidism may be palpable in labium majora or inguinal canal. When benign and malignant tumors occur in the gonads of CAIS, mild fullness or masses can be palpable in adnexal, inguinal, or labial regions (24). The testis can be asymmetric such as one at the inguinal region and the other in the abdomen (51).

## Diagnosis and evaluation

CAIS diagnosis can be made prenatal, in childhood, during puberty, and at adulthood (52–55). In fact, CAIS patients with intraabdominal gonads are readily overlooked at birth or during childhood due to the typical female phenotype and absence of typical symptoms (48). Prepubertal diagnosis is challenging and can be suspected with labial swelling or bilateral inguinal masses or if testis is found during inguinal hernia repair (13, 52, 56). A diagnosis of CAIS during puberty is similar to that of PA with additional evaluation. PA can be caused by various functional (e.g., hypogonadotropic hypogonadism) and anatomical factors (e.g., absence of uterus as in CAIS) (57). When bilateral inguinal hernia was examined during infancy or PA coexisted with elevated T levels without signs of virilization, CAIS should be considered (13). As in the diagnosis of other DSD, a targeted and structured approach is suggested in prompt and precise evaluation of CAIS, including clinical, laboratory, imaging, genetic assessment, and sometimes gonadal biopsy (58).

## Biochemical assessment

Endocrine hormone assay: Assay of reproductive endocrine hormones is frequently used to identify HPGA and the potential causes of amenorrhea, which is also helpful for the diagnosis of CAIS. Tests of FSH, LH, E, T, and AMH facilitate to evaluate the function of HPGA, reason of PA, and differential diagnosis of CAIS. For pituitary gonadotropin, FSH level may be in the normal range while LH may be slightly above the normal upper limit of men owing to distinguished feedback on FSH and LH (13). For sex hormones, the E level is within normal male ranges and lower than normal female values which is opposite to T which is within normal male values and higher than normal female ranges (13). AMH, the most useful marker of functional testicular tissue, is normal or higher in CAIS as well as in unilateral cryptorchidism than in bilateral ectopic testis (59). The combination of relatively high levels of serum T and LH and lack of axillary/pubic hair is a useful clue for CAIS, as other laboratory tests for a definitive diagnosis of CAIS such as genetic test, AR mutation, and androgen binding capacity are not universally available and have a high cost.

HCG stimulation test: After HCG stimulation (>100 ng/dL), a markedly elevated T level is indicative of the presence of testicular tissue, while the ratio of T to Δ4-androstenedione (Δ4A) increases and the ratio of T to DHT decreases (60).

Chromosomal karyotype testing: Cytogenetics for karyotype is important to identify the genetic basis as 24.7% patients with PA had abnormal karyotype, among which 29.8% harbored a 46, XY karyotype which is consistent with CAIS (61).

## Medical imaging tests

Pelvic imaging studies can demonstrate the absence of uterus, cervix, ovaries, and part of the vagina (62). Furthermore, for cases with unpalpable testis, pelvic ultrasound can be used to detect undescended testis, with magnetic resonance imaging (MRI) being of greater sensitivity and specificity (63). To be noteworthy, it is important for radiologists to be familiar with the disorder for correct clinical judgment on imaging findings (49). Testicular parenchyma in 65% CAIS was heterogenous on MRI with paratesticular cyst or low-signal-intensity, well-defined mass (64). Dual-energy X-ray absorptiometry (DXA) can be used to detect bone mineral density.

## Molecular genetic testing

A definitive diagnosis of CAIS depends on the detection of AR mutation or androgen binding capacity. Molecular genetic etiology can be determined in the majority of CAIS cases, whereas other factors may be involved in AIS as AR mutation is not always identified in CAIS cases, and phenotype–genotype correlation in cases with AR mutations is still unclear as the identical *AR* mutation (p.P914S) can be detected in both CAIS and PAIS with different phenotypes (4, 26, 65). In addition, AR mutation is found not just in cases with CAIS but also in prostate cancer and Kennedy’s syndrome (66). It is universally accepted that similar female phenotypes in CAIS are overlapped with differential diagnosis caused by a completely distinct molecular change, so molecular genetic testing of AR is beneficial for a definitive molecular diagnosis and indicated for otherwise undetermined cases (26). Compared to PAIS, AR mutation is easily detected in CAIS patients (67).

## Gonadal pathology

In CAIS, the gonads are abnormally located testes of degeneration and dysgenesis with delayed germ cell development and prolonged expression of specific markers including placenta-like alkaline phosphatase (PLAP) (68). Leydig cell hyperplasia, atrophic embryonal-type seminiferous tubules with Sertoli cells, increased fibrosis, incomplete spermatogenesis, and incidental spermatogonia may be observed in cryptorchidectomy specimen without neoplasia (4, 48, 69). Both benign and malignant tumors can be detected in specimens after orchiectomy. Although most tumors are benign, such as paratesticular cysts which may be remnants of mullerian structure and Sertoli cell adenoma, premalignant and malignant changes can be revealed with an increasing presence compared to that in a normal population (64). Paratesticular leiomyoma and significant hyperplasia of Sertoli cells have been reported in a 16-year-old girl (51). Albeit the exact gonadal tumor rates are inconsistent and heterogeneous (0.8%–22%), it is widely accepted that the risk increases with age (4, 70). Sex cord/stromal neoplasia and mesenchymal tumors could be detected in the excised gonadal tissue simultaneously (42).

## Differential diagnosis

CAIS should be differentiated from other PA-related etiology, such as Swyer syndrome, Mayer–Rokitansky–Küster–Hauser (MRKH) syndrome, Leydig cell hypoplasia (LCH), steroidogenesis enzymatic deficiency such as 5α-reductase deficiency (5α RD), 17β-hydroxysteroid dehydrogenase-3 (17β-HSD-3) deficiency, and p450 oxidoreductase deficiency (PORD) (24, 38) (shown in **Table 1**).

**Table 1:** Similarities and differences of clinical and laboratory aspects among CAIS, Swyer syndrome, MRKH syndrome, and LCH.

disorders clinical aspects	CAIS	Swyer syndrome	MRKH syndrome	LCH
Primary amenorrhea	+	+	+	+
Breast development	+	_	+	_
Axillary/pubic hair	–	_	+	+/-
External genitalia	Female	Female	Female	Female
Uterus	_	+	_	_
Gonads	Testes	Streak gonads	Ovaries	Testes
FSH	N	+	_	N
LH	+	+	_	+
T	N^a^	N	N	_
E	N^b^	_	+	_
Androgen receptor	+	_	_	_
Chromosome karyotype	46, XY	46, XY	46, XX	46, XY
Risk of gonadal tumors	+	+	_	+
Vaginoplasty	_	_	+	_

N, normal; CAIS, complete androgen insensitivity syndrome; MRKH syndrome, Mayer Rokistansky Küster Hauser syndrome; LCH, Leydig cell hypoplasia; FSH, follicle-stimulating hormone; LH, luteinizing hormone; T, testosterone; E, estrogen.

a Donates normal male range (>female values).

b Donates normal male range (<female values).

**Figure 2:**
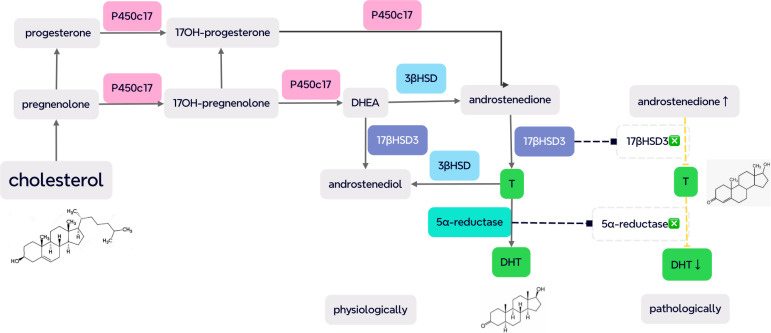
Several key enzymes in the steroidogenesis of testosterone (T) and dihydrotestosterone (DHT), including P450c17, 3β hydroxysteroid dehydrogenase (3βHSD), 17β hydroxysteroid dehydrogenase 3 (17βHSD3), and 5α-reductase. On 17βHSD3 deficiency, the T/androstenedione (Δ4A) value decreases (<0.8) with increased T/DHT (>20), indicative of 5α RD due to an elevated level of substrates and the low concentration of products (pathologically demonstrated as dash lines) [from (60) and (80)].

1. Swyer syndrome (46, XY complete/pure gonadal dysgenesis) is also a rare congenital DSD with an estimated proportion of 1:80,000 to 100,000 births and whose etiology is undetermined (71). Swyer syndrome may be caused by SRY mutation (10%–15%), FTHL17, STARD8, SOX9, MAP3K1, NR5A1, and desert hedgehog gene (DHH) mutation as well as other unidentified genes (71, 72). Underdeveloped and undifferentiated streak gonads do not produce T and AMH which, in turn, leads to regression of wolffian ducts and the development of müllerian ducts to uterus, fallopian tubes, and upper vagina. Albeit lacking both fetal E and T, the lower vagina is formed under the influence of maternal and placental E (71). Hence, cases with Swyer syndrome have intraabdominal streak gonads (neither testis nor ovaries) with female external and internal genitalia. Although both Swyer syndrome and CAIS can present with PA and increased risk of gonadal tumors due to the presence of Y chromosome, cases with Swyer syndrome may complain of delayed puberty, different from that in CAIS, and have their own hormonal characteristics due to the dysfunctional gonads, including increased FSH and LH levels and decreased E, T, and AMH concentrations (71).
2. MRKH syndrome is a rare congenital müllerian duct agenesis with underdevelopment or complete absence of the uterus and upper part of the vagina in a 46, XX female patient with ovaries and normal female sex characteristics with an estimated prevalence of 1 in 5,000 live female births (73–75). The mechanism is still unclear, although 16p11.2 microdeletions may be involved in the congenital genital abnormalities (73). PA and infertility are also the primary complaints (75). Sex hormone assays are commonly within normal female ranges. Physical examination of axillary/pubic hair and chromosomal test are the main differential issues between MRKH and CAIS (shown in **Table 1**).
3. LCH is a rare autosomal recessive DSD caused by LH/chorionic gonadotropin receptor gene (LHCGR) mutation in phenotypically female individuals with 46, XY karyotype and cryptorchidism. With the presence of AMH, the müllerian ducts regressed. Uterine PA and absence of thelarche during puberty can be observed in such cases. Typical LCH cases present a poor response to HCG stimulation (1,500 U/d*3d) with high LH and AMH, normal or high FSH, and low E and T levels (76–78). Before testing of a specific pathogenic gene, a comprehensive clinical analysis is particularly important for similarities with different congenital abnormalities (79).
4. Steroidogenic enzymatic deficiency: T and its metabolite DHT play important roles in the differentiation and development of male internal and external genitalia (76). Thus, enzymatic deficiencies involving the synthesis of T and DHT frequently lead to DSD, such as 5α RD, 17β-HSD-3 deficiency, PORD, and 3 beta-hydroxysteroidal deficiency which should be considered in the differential diagnosis of CAIS (80–82). HCG stimulation test can be applied to help determine specific enzymatic deficiencies, such as T/Δ4A value decreases indicative of 17β-HSD-3 and T/DHT increases suggestive of 5α RD (60) (shown in **Figure 2**).
5. As both CAIS and some severe PAIS are caused by AR mutation and can be assigned as females albeit with male karyotype, they should be differentiated as well. Clitoromegaly at birth and pubic/axillary hair during puberty may be present in PAIS cases due to residual AR activity which needs careful examination by specialists (60). It should be emphasized that structured step-by-step workup algorithm by highly specialized medical teams is crucial to accurately evaluate the external genitalia and form the first impression of the underlying cause (83).

In a nutshell, for precise diagnosis and differentiation of CAIS, thorough examination of breast development, body hair, and labial and inguinal regions, endocrinological hormone assay, chromosomal karyotypes, and imaging findings of presence or absence of müllerian duct derivatives are all required. To confirm the clinical impression after thorough evaluation and clinical reasoning, corresponding molecular detection and androgen binding test can be performed.

## Management of CAIS

Optimal management for CAIS necessitates multidisciplinary collaboration including pediatric endocrinologists, gynecologists, urological physicians, psychological experts, genetic counsellors, and even experts on transplantation. To obtain good clinical outcomes, the risks and benefits of retention or removal of gonads and different hormone supplement therapies should be thoroughly evaluated. Several issues with unique challenges are discussed in details as follows, including timing of gonadectomy, hormonal supplement strategy, pregnancy, genetic counselling, and its psychological effect and education (14, 62).

## Surgical treatment

### Preservation or removal of gonads and timing of gonadectomy

Tumor risks in undescended testis are appropriately 25% (benign) and 3%–10% (malignant) which is negligible for children and adolescent CAIS patients (<1%). Thus, it is recommended that testis be preserved until puberty onset and maturation based on current epidemiological and histological data (62). However, the risk increases with ages which can achieve 33% at 50 years of age (13). Most gonadal tumors in CAIS were postpubertal or in adulthood with very rare cases in prepuberty, which can be germ cell neoplasia *in situ* (GCNIS), and gonadectomy is suggested to be postponed until 25 years old, which is different from the management of other DSD patients with Y chromosome (70, 84). According to a study of DSD for 20 years, gonadectomy can be performed until or after puberty in CAIS, distinguished from that in Swyer syndrome because tumors were detected after 18 years of age (85). In recent years, it is suggested that shared decision-making is adopted between patients and their families if gonads are preserved until adulthood (63, 70). For patients who underwent removal of testis, laparoscopic gonadectomy was frequently safe without conversion to laparotomy (86). Several tumor markers can be used in the detection of GCNIS, including PLAP and SCF (stem cell factor) with the limitation of testicular tissue samples applicable only (13). In addition, yolk sac tumor has been reported in infancy (53).

For patients who refuse orchiectomy, periodical imaging examination is suggested to detect gonadal tumors early, but even MRI may fail to detect GCNIS (13). For labioscrotal or inguinal testis, annual follow-up by ultrasonography is recommended, with MRI being more preferrable for intraabdominal gonads (4).

Vaginoplasty: According to a retrospective study of 29 patients with CAIS, 70% (7/10) were sexually active in postpubertal patients with vaginoplasty versus 80% (12/15) in cases without vaginal reconstruction, so vaginoplasty is not recommended in CAIS (87). However, the less invasive vaginal dilatation can be performed in some cases (13).

## Hormone replacement therapy

E supplement is usually not necessary for CAIS cases with testis preservation until post-pubertal stage when they commonly present with spontaneous breast development due to E aromatized from T. However, gonadectomy may be undertaken at childhood in some cases as a result of bilateral inguinal “hernia”. In such cases, the lack of either kind of gonads and deficiency of both male and female sex hormones frequently lead to poor breast development. In CAIS patients with bilateral orchiectomy during adulthood to avoid or treat gonadal malignancy, E production derived from T will decrease and mitigate the protective action on bone, metabolism, cognition, and cardiovascular health, probably similar to that in normal postmenopausal women. Thus, hormone replacement (HR) is strongly recommended to stimulate secondary female sexual characteristics and prevent E-deficiency-associated complications which can be initiated at 11 to 12 years of age (4, 13).

Timing, doses, hormonal type, and mode of administration in HR are dependent on the patients’ age, retention or removal of gonad, and response and compliance to the therapy. The root of HR is initiation from low doses with gradual escalation to maintenance therapy. No consensus exists on the initiative dose of E, with 2–5 ug/day or 50–100 ng/kg suggested and periodical adjustment in the next 2 to 3 years in order to reach the normal adult dose. Excessive E supplement may result in premature closure of epiphysis and has a negative impact on the eventual stature. The current suggested maintenance dose of E is daily at 1 to 2 mg oral E or 40–50 ug transdermal E (4, 13). With the absence of uterus, progestin is usually unnecessary, unlike that in postmenopausal women, to protect the endometrium. T and selective estrogen receptor modulator were also reported with a less frequency in HR (60). E at 1.5 mg/day or T at 50 mg/day has been reported in adult CAIS cases which demonstrated equivalent mental health-related QOL and psychological well-being, whereas T was superior to E in improving sexual desire (88).

## Pregnancy and lactation

Owing to lack of uterus, CAIS couples cannot conceive spontaneously but may expect their own child through adoption or surrogacy. For cases with desire for breastfeeding, induced lactation can even be achieved by the combination of pharmacological and non-pharmacological therapies. Estrogen therapy, galactagogues, domperidone, and mechanical breast stimulation were reported for the preparation of breastfeeding 1 or 2 months before the birth of the child to mimic physiological breast development and lactation (89, 90). Although it was insufficient for effective long-term breastfeeding, small, unquantified milk could be secreted in such cases, allowing partially successful breastfeeding for the untraditional mother (89, 90).

## Genetic counselling

Familial screening is useful for detecting asymptomatic patients and carriers in the proband’s relatives and providing information for genetic counseling of their offspring. Theoretically, a prenatal consideration of CAIS can be achieved by the combination of chromosomal karyotype assessment from amniocentesis or fetus-free DNA testing from maternal blood and genital sex identification through prenatal ultrasonography (48). As it is inherited at an X-chromosomal recessive manner, for maternal carriers of AR mutation, there is a 50% chance of birth of CAIS patients in genetically men and CAIS carriers in karyotypically female individuals and a 50% possibility of a healthy baby regardless of the biological gender. For the proband’s siblings, 46, XX individuals all present no similar abnormalities with 50% AR mutation carriers, and half of the 46, XY individuals may be affected.

## Psychological support and education

Karyotypically male cases with CAIS are usually raised as girls and require no gender reassignment after a definitive diagnosis due to phenotypically female appearance (91). Patients with CAIS experience both disease-related and treatment-related physical and psychological stress, and psychological support is a key factor in the management of DSD besides medical intervention (92). It is reported that CAIS cases had an increased psychiatric morbidity (93). Gonadectomy in CAIS may have a negative impact on psychological wellbeing and sexual satisfaction (88). On one hand, it is suggested to postpone orchiectomy to ensure the patients’ maturity and autonomy considering the relatively low risk and potential benefit of prevention of psychological and physical trauma associated with surgery (87). On the other hand, it is helpful to provide education and psychological support to the patients and their families (48). After proper intervention, the QOL of these patients with DSD may not be significantly affected according to a previous report (94). Now it is considered reasonable to reveal the influence of the disorder and current medical intervention in an age-appropriate manner and provide reasonable suggestions of family building and fertility (60). Access to expert DSD care teams, patients and family education, psychological support including acceptance, inclusivity, and shared decision-making are important to help them encounter challenges and uncertainties associated with the disease and improve the patients’ QOL (91).

## Future direction

The prompt and accurate diagnosis of CAIS is still a clinical challenge especially in some emergent conditions as delayed definitive identification and misdiagnosis as strangulated femoral hernia has been reported, albeit the increasing knowledge and more reported cases regarding such a rare condition. Hence, comprehensive education may be required to ensure that relevant clinicians as well as radiologists can consider the entity in suspected cases. Timely recognition of gonadal tumors calls for sensitive and specific biomarkers which need further investigation. Although approximately 95% of individuals with CAIS can be identified with a molecular diagnosis, the remaining type II of AR gene mutation-negative group of patients remains elusive. It may be influenced by detection techniques or epigenetic repression or the abnormal upstream or downstream gene of AR pathway, which needs to be further investigated and perhaps can shed light on other causative molecular pathogenesis of CAIS. As an X-linked recessive monogenic disorder, theoretically, gene therapy has a promising therapeutic potential in the management of CAIS.

## Summary

CAIS is a rare DSD caused by AR gene, located on chromosome Xq1.1-1.2, the mutation of which leads to the absence of both mullerian and wolffian duct derivatives and presents with female external genital phenotype in a 46, XY individual with testis as a result of AR defect and consequent T dysfunction. Clinical manifestations include bilateral inguinal “hernia” or labial swelling in childhood and PA and infertility during puberty and adulthood. The main concern lies in definitive diagnosis owing to its rarity, lack of specific presentations, and readily overlooked property. In phenotypic female cases with bilateral inguinal mass or pubertal thelarche and increased serum T without signs of virilization, a diagnosis of CAIS should be suspected with differentiation form Swyer syndrome, MRKH syndrome, LCH, PAIS, and steroidogenic enzymatic deficiencies. The management of CAIS includes psychological support and education, retention or removal of gonads, HR, and genetic counseling.
